# Novel Advancements in COVID-19 and Neuroscience

**DOI:** 10.3390/jpm14020143

**Published:** 2024-01-28

**Authors:** Alessio Simonetti, Evelina Bernardi, Gabriele Sani

**Affiliations:** 1 Department of Neurosciences, Sensory Organs and Chest, Section of Psychiatry, Fondazione Policlinico Universitario Agostino Gemelli IRCCS, 00168 Rome, Italy; evelinabernardi@gmail.com (E.B.); gabriele.sani@unicatt.it (G.S.); 2 Menninger Department of Psychiatry and Behavioral Sciences, Baylor College of Medicine, Houston, TX 77030, USA; 3 Department of Neurosciences, Sensory Organs and Chest, Section of Psychiatry, Università Cattolica del Sacro Cuore, 00168 Rome, Italy

From an initial cluster of cases reported in Wuhan, the SARS-Cov-2 infection has since spread globally, causing a pandemic that began on 11 March 2020 [1,2]. In the following years, researchers’ efforts to define the COVID-19 syndrome and its sequelae produced more than 200,000 papers, providing a steep increase in knowledge around its pathophysiology [3,4], clinical characteristics [5,6], and treatment [7,8,9]. The most evident feature of the COVID-19 syndrome resides in its variability; the clinical presentations of acute SARS-CoV-2 infection span from asymptomatic to severely symptomatic forms, which can lead to death due to pneumonia, acute respiratory distress syndrome (ARDS), and multiple organ failure [10,11,12,13].

Furthermore, one of the earliest and most striking finding was that physical distress brought about by COVID-19 does not generally extinguish with the end of the infection [14,15,16]. A substantial amount of people have reported experiencing clinical sequelae in the weeks or months following symptom onset. These symptoms, known as post-COVID-19 syndrome, or long-COVID-19 syndrome, include cough, dyspnea, sleep disorders, gastrointestinal complaints, musculoskeletal problems, and neurological and psychiatric issues [17,18,19,20,21]. Neuropsychiatric symptoms have been the subject of extensive research during the COVID-19 pandemic because of the high prevalence of anxiety, depression, psychosis, dementia, and cognitive disturbances during and after SARS-CoV2 infection [22,23,24,25,26,27,28,29]. Even though several hypotheses have gradually been ruled out to disentangle the pathophysiology linking acute infection, physical sequelae, acute and chronic symptoms, and psychopathology [30,31,32,33], several aspects of the COVID-19 syndrome’s neuropsychiatric features remain undiscovered [34], and the need to define the population at risk is still unmet [35,36]. Therefore, the present Special Issue pursued to provide a comprehensive analysis of the various aspects of COVID-19-associated acute and chronic neuropsychiatric features.

As demonstrated by the umbrella review made by Sampogna and colleagues, anxiety and depression represent one of the most evident manifestations of COVID-19-reated psychopathology, with rates ranking up to 23.5% [37]. Specific forms of depression, such as depression with excitatory symptoms, also called mixed depression, have been associated with a high risk of suicide and a greater number of physical symptoms than depression without excitatory symptoms in subjects with post-COVID-19 syndrome [38]. COVID-19 has also been associated with new-onset psychosis, as shown by Moccia and colleagues. Clinical features of COVID-19-associated psychosis, like those observed in the context of several infectious diseases, include higher age at onset and response to a low-to-moderate dose of antipsychotics, as well as a quicker recovery and a generally favorable prognosis [39]. The authors proposed that viral entry in brain structures may facilitate the onset of psychotic symptoms in vulnerable persons. 

In this Special Issue, the aforementioned psychopathological manifestations have also been explored in relation to lifespan. Serra and colleagues reported that consultations at the emergency departments for suicidal ideation in youth significantly increased from the pre-pandemic period to the end of the pandemic [40]. With regard to specific suicide attempts, self-poisoning incremented dramatically, possibly due to self-medication or dysfunctional self-regulation attitudes. On the other hand, Cipriani and colleagues and Janiri and colleagues investigated the effect of hospitalization on the mental health of elderly people. Their findings revealed that subjects who were 70 years of age or older were at a higher risk of psychiatric symptoms and delirium compared to younger subjects [41]. Additionally, women aged more than 65 years showed more psychiatric symptoms and few resilience factors than men after COVID-19 infection, even though low resilience levels significantly predicted psychological distress in both men and women [42]. The effect of COVID-19 on women’s mental health was further explored by De Chiara and colleagues. Pregnant women during the pandemic period were found to more likely have thoughts of harming themselves; moreover, the pregnant female participants showed higher prevalence rates of clinically relevant maternal depression and anxiety compared to those who delivered amidst the pandemic, adding to the stress factors that impair the normal processes of maternal care and emotional stability [43].

Novel advances presented in this Special Issue are not limited to classic psychiatric syndromes and include alterations of functional psychopathology, neurology, and brain structure and function. To this extent, the effect of COVID-19 has been investigated in subjects with functional movement disorders (FMDs), i.e., a spectrum of psychosomatic symptoms particularly sensitive to stress [44]. Subjects with FMDs reported higher levels of psychological distress during the COVID-19 pandemic, as well as increased emotional dysregulation and cyclothymic traits. Cyclothymia is a locked-in stable predisposition to rapid and spontaneous mood fluctuations and over-reactivity to external and internal stimuli [45,46,47]. Difficulties in emotional regulation may prevent cyclothymic individuals from regulating their feelings against stressful stimuli in a balanced way [48,49]. This can cause rapid and unexpected mood swings in response to stress, including the one caused by COVID-19. Perrottelli and colleagues reviewed the effect of COVID-19 on brain function, as deduced by the COVID-19-related “brain fog” and other cognitive impairment commonly observed after COVID-19 infection. The available evidence revealed the presence of impairment in executive functions, processing speed, attention, and memory [50]. As proposed by Oner and colleagues, cognitive impairment may be related to high homocysteine levels [51], which plays a role in cognitive impairment, memory decline, and brain damage [52]. Finally, Kotzalidis and colleagues systematically reviewed the literature concerning brain alterations in post-traumatic stress disorder (PTSD) and post-COVID-19 syndrome through neuroimaging techniques. They found that brain PTSD and post-COVID-19 syndrome share several common brain alterations, such as hypometabolism in the insula and caudate nucleus, reduced hippocampal volumes, subarachnoid hemorrhages, and white matter hyperintensities [53]. 

In summary, COVID-19 appears to exert a deep effect on psychopathology, as well as brain structure and function. Neuropsychiatric alterations brought about by COVID-19 can appear during, and be sustained after, SARS-CoV2 infection, in addition to affecting people at their earlier or later stages in life. The purpose of this Special Issue resides in fostering further research to ultimately disentangle the pathophysiology underlying COVID-19. This may lead to the discovery of personalized treatments [54,55,56], which might reduce illness burden and increase well-being. To pursue such an aim, further research, as well as greater effort, is still needed. 
